# Genome-wide functional genetic screen with the anticancer agent AMPI-109 identifies PRL-3 as an oncogenic driver in triple-negative breast cancers

**DOI:** 10.18632/oncotarget.7462

**Published:** 2016-02-17

**Authors:** Hamid H. Gari, Christy M. Gearheart, Susan Fosmire, Gregory D. DeGala, Zeying Fan, Kathleen C. Torkko, Susan M. Edgerton, M. Scott Lucia, Rahul Ray, Ann D. Thor, Christopher C. Porter, James R. Lambert

**Affiliations:** ^1^ Department of Pathology, University of Colorado School of Medicine, Aurora, CO, USA; ^2^ Department of Pediatrics, University of Colorado School of Medicine, Aurora, CO, USA; ^3^ Department of Medicine, Boston University School of Medicine, Boston, MA, USA

**Keywords:** AMPI-109, PRL-3, triple-negative breast cancer, phosphatase, functional genomics

## Abstract

Triple-negative breast cancers (TNBC) are among the most aggressive and heterogeneous cancers with a high propensity to invade, metastasize and relapse. Here, we demonstrate that the anticancer compound, AMPI-109, is selectively efficacious in inhibiting proliferation and inducing apoptosis of multiple TNBC subtype cell lines as assessed by activation of pro-apoptotic caspases-3 and 7, PARP cleavage and nucleosomal DNA fragmentation. AMPI-109 had little to no effect on growth in the majority of non-TNBC cell lines examined. We therefore utilized AMPI-109 in a genome-wide shRNA screen in the TNBC cell line, BT-20, to investigate the utility of AMPI-109 as a tool in helping to identify molecular alterations unique to TNBC. Our screen identified the oncogenic phosphatase, PRL-3, as a potentially important driver of TNBC growth, migration and invasion. Through stable lentiviral knock downs and transfection with catalytically impaired PRL-3 in TNBC cells, loss of PRL-3 expression, or functionality, led to substantial growth inhibition. Moreover, AMPI-109 treatment, downregulation of PRL-3 expression or impairment of PRL-3 activity reduced TNBC cell migration and invasion. Histological evaluation of human breast cancers revealed PRL-3 was significantly, though not exclusively, associated with the TNBC subtype and correlated positively with regional and distant metastases, as well as 1 and 3 year relapse free survival. Collectively, our study is proof-of-concept that AMPI-109, a selectively active agent against TNBC cell lines, can be used as a molecular tool to uncover unique drivers of disease progression, such as PRL-3, which we show promotes oncogenic phenotypes in TNBC cells.

## INTRODUCTION

Breast cancer is a heterogeneous disease exhibiting diverse biological characteristics and clinical responses. Gene expression profiling has defined signature clustering of at least five molecular subtypes including an aggressive form known as triple-negative breast cancer (TNBC) [1, 2]. TNBCs are typically high-grade (poorly differentiated) and rapidly progressive, with lower survival than the remaining subtypes [3]. By definition, TNBCs fail to express three receptors shown to promote many breast cancers: estrogen receptor, progesterone receptor, and human epidermal growth factor receptor 2. For many patients with breast cancer targeting of these molecules significantly improves outcome [4]. Because TNBCs lack expression of these targets and other molecular targets have not been identified, cytotoxic chemotherapies are most frequently utilized [5, 6]. These treatments are limited by poor long-term therapeutic response, non-selective toxicities, and clonal progression of disease with the development of resistance.

In an attempt to identify molecular-based therapies for TNBC, extensive transcriptomic analysis of large TNBC datasets has been carried out [1, 7, 8]. Gene clustering analysis has now defined at least six molecular subtypes of TNBC, including: two basal-like, a mesenchymal, a mesenchymal stem-like, an immunomodulatory, and a luminal androgen receptor subtype [7]. The identification of these subtypes may aid in elucidation of signaling pathways that can potentially be targeted in drug discovery efforts.

Numerous epidemiological studies have demonstrated the importance of vitamin D, dietary or otherwise, in preventing various cancers [9]. Additionally, the therapeutic potential of 1α,25-dihydroxyvitamin D_3_ (1,25D), the biologically active metabolite of vitamin D, and its analogs in cancer is well-documented [10]. However, the inherent calcemic toxicity of vitamin D, particularly during daily dosing regimens, has prevented its general use as an anticancer agent. Thus, the development of vitamin D analogs exhibiting potent antiproliferative activity but reduced systemic toxicity has become an active area of research.

**Figure 1:**
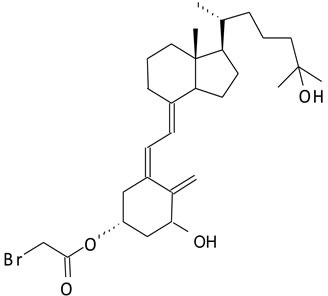
Structure of AMPI-109

We have developed novel analogs of vitamin D and its pre-hormonal form, 25-hydroxyvitamin D_3_, that specifically bind and label the ligand-binding pocket of the nuclear receptor for vitamin D (vitamin D receptor, VDR) [11-14]. Previously, we reported the synthesis and antitumorigenic properties of a novel compound, AMPI-109 (1α,25-dihydroxyvitamin D_3_-3-bromoacetate) (Figure 1) [15-17]. AMPI-109 was originally synthesized as a biochemical tool to probe the residues of VDR that contact the hormone [13]. More recently we have investigated the antiproliferative effects of AMPI-109 in a variety of cancer types including prostate, pancreas and kidney [16-18]. In these studies AMPI-109 exerted significant antiproliferative and proapoptotic activities and was more potent than vitamin D in doing so.

Here, we examined the antitumorigenic properties of AMPI-109 in human breast cancer cells. AMPI-109 potently induced apoptosis in TNBC cells of various molecular subtypes, yet had little to no effect on the majority of non-TNBC and non-tumorigenic cell lines examined. To gain a mechanistic understanding of AMPI-109 specific action in TNBC cells and towards identifying molecular alterations unique to TNBC, we carried out a genome-wide functional shRNA screen in the TNBC cell line, BT-20, to identify genes that, when silenced, conferred resistance to AMPI-109. The highest ranking hit in our screen was *PTP4A3* encoding Phosphatase of Regenerating Liver (PRL-3).

In this report, we demonstrate that reduction of PRL-3 expression or impairment of PRL-3 catalytic activity leads to substantial growth inhibition and a reduction in the migratory and invasive ability of TNBC cells, partially phenocopying the effects of AMPI-109. In a retrospective study, we show that PRL-3 is more highly expressed in TNBC relative to other breast cancer subtypes, and that PRL-3 expression associates with the presence of regional disease and distant metastases. Because the vast majority of TNBC deaths result as a consequence of metastatic disease to visceral organs, new therapies targeting the PRL-3 signaling axis could have significant impact in reducing the mortality associated with TNBC.

## RESULTS

### AMPI-109 impairs TNBC cell proliferation and induces apoptosis

**Figure 2:**
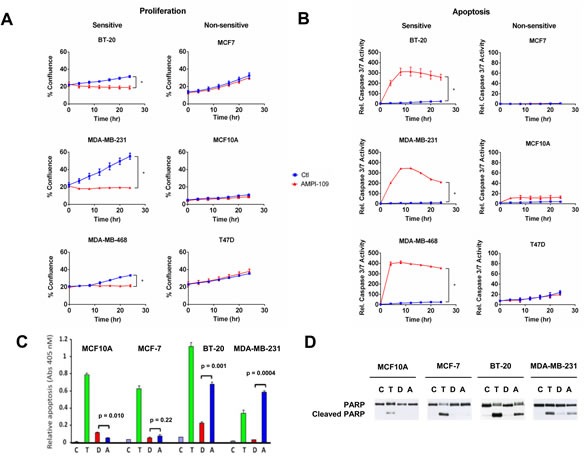
AMPI-109 mediated growth inhibition and induction of apoptosis is specific for TNBC cell lines **A.** The response of the indicated TNBC (BT-20, MDA-MB-231 and MDA-MB-468) and non-TNBC (MCF7, MCF10A and T47D) cell lines to ethanol vehicle control (Ctl; blue) or 100 nM AMPI-109 (AMPI-109; red) was determined by real time kinetic monitoring of cellular proliferation. **B.** The same cell lines were analyzed for induction of apoptosis by real time kinetic monitoring of caspase 3/7 activity after treatment with ethanol vehicle control (Ctl; blue) or 100 nM AMPI-109 (AMPI-109; red). For (A and B) * = *p*-value < 0.05 as determined by Student t test on last time-point. **C.** the indicated cell lines were treated with ethanol control (C; purple), paclitaxel (10^−7^ M, (T; green)), 1,25D (10^−7^ M, (D; red)), or AMPI-109 (10^−7^ M, (A; blue)) for 24 hrs. Cytoplasmic nucleosomal DNA was measured by ELISA to assess apoptosis. **D.** The same cells as in **C.** were treated for 24 hrs and PARP cleavage determined by immunoblot.

We examined the ability of AMPI-109 to inhibit the proliferation of breast cancer cells of various molecular subtypes including TNBC. A cohort of 12 breast cancer cell lines was treated with AMPI-109 at its approximate IC_50_ value of 100 nM (determined by cellular proliferation assays in response to escalating doses of AMPI-109) or vehicle control. Of the 7 cell lines that showed significant response to AMPI-109, 6 were TNBC cell lines representing 5 different molecular subtypes of TNBC (Table 1 and Figure 2A). In these experiments we also compared AMPI-109 to its parent compound, 1,25D. AMPI-109 was far superior to 1,25D in inhibiting the growth of all cell lines tested (Table 1). Importantly, AMPI-109 had no effect on proliferation of non-tumorigenic breast cells (MCF10A) or the majority of non-TNBC cell lines (Table 1 and Figure 2A).

**Table 1:** AMPI-109 inhibits growth of multiple TNBC subtypes

Cell Line	Subtype	1,25D % Control	AMPI-109 % Control	1,25D: AMPI-109 p-value
MCF10A	Nonmalignant	102	99	0.23
MCF7	Luminal A	95	93	0.27
T47D	Luminal A	98	94	0.40
BT-474	Luminal B	87	**66**	<0.0001
MDA-453	HER2	94	86	0.26
SKBR3	HER2	100	99	0.89
MDA-MB-231	TN (MSL)	96	**44**	<0.0001
MDA-MB-468	TN (BL1)	86	**39**	<0.0001
BT-20	TN (Unclass.)	82	**29**	<0.0001
BT-549	TN (M)	94	**69**	<0.0001
HCC70	TN (BL2)	83	**49**	<0.0001
SUM149	TN (BL2)	92	**39**	<0.0001

The cell lines were treated for 36 hours with AMPI-109 (100 nM), 1,25D (100 nM) or ethanol control and MTS assay performed. P values represent significance between 1,25D and AMPI-109. TN, triple-negative; MSL, mesenchymal stem like; BL1, basal like 1; Unclass., unclassified; M, mesenchymal, BL2, basal like 2.

To examine the ability of AMPI-109 to induce apoptosis, we performed real time kinetic imaging of caspase 3/7 activity. We observed a strong induction of apoptosis in the TNBC cell lines, but not in the non-TNBC cell lines in response to AMPI-109 (Figure 2B). We also performed ELISA assays to detect cytoplasmic nucleosomal DNA and immunoblot analysis for cleavage of poly (ADP)-ribose polymerase (PARP). Interestingly, paclitaxel, a commonly used drug in breast cancer, induced apoptosis and PARP cleavage in all cells tested whereas AMPI-109 specifically induced apoptosis in TNBC cells (Figure 2C-2D). Taken together, these results indicate that AMPI-109 preferentially impairs the survival ability of TNBC cells and that AMPI-109 is more efficacious than 1,25D in doing so.

### The ability of AMPI-109 to block TNBC growth is independent of VDR

**Figure 3:**
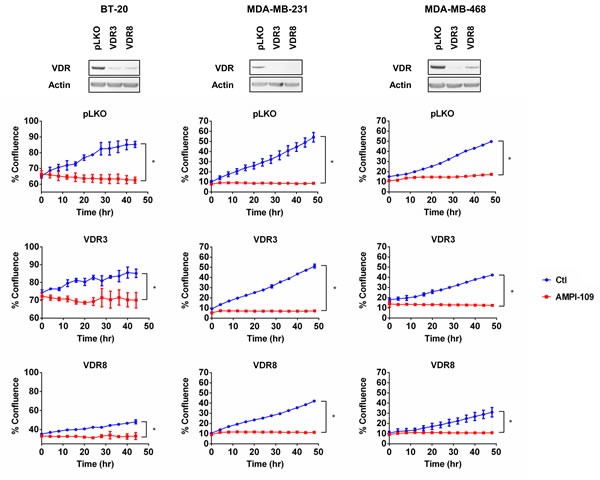
AMPI-109 action is independent of VDR (Top) Immunoblot analysis of VDR levels in two pools of BT-20, MDA-MB-231 and MDA-MB-468 cells with VDR shRNAs (VDR3 and VDR8). pLKO is non-silencing control. (Bottom) Corresponding graphs depict response of the indicated pools of VDR pLKO and shRNA cells to ethanol vehicle control (Ctl; blue) or 100 nM AMPI-109 (AMPI-109; red) as determined by real time kinetic monitoring of cellular proliferation. * = *p*-value < 0.05 as determined by Student t test on last time-point.

1,25D elicits its antiproliferative effects on cancer cells through binding to the ligand-activated transcription factor, VDR. As described above, AMPI-109 was originally developed as a probe to identify which residues of the VDR ligand binding domain contact the hormone [13]. Because AMPI-109 is an analog of 1,25D, a key mechanistic question is whether it, like 1,25D, exerts its antitumorigenic properties through binding to VDR. We used shRNAs directed against VDR in three TNBC cell lines and examined their response to AMPI-109. Real-time kinetic monitoring of cellular proliferation following treatment with AMPI-109 was performed in TNBC cell lines exhibiting knock down of VDR (Figure 3). AMPI-109 was equally effective at inhibiting the growth of control cells and cells with VDR knock down demonstrating that AMPI-109 exerts its antiproliferative effects through a non-VDR dependent mechanism (Figure 3). We emphasize that all of our data examining the role of VDR in AMPI-109 action, and in comparing AMPI-109 to 1,25D, discussed above, indicate that AMPI-109 is acting through a novel mechanism, distinct from 1,25D and VDR. Thus, despite their related chemical structures, AMPI-109 elicits pro-apoptotic actions in TNBC cells through a mechanism independent of VDR.

### Functional genomic screen identifies PRL-3 as a modifier of AMPI-109 action

**Figure 4:**
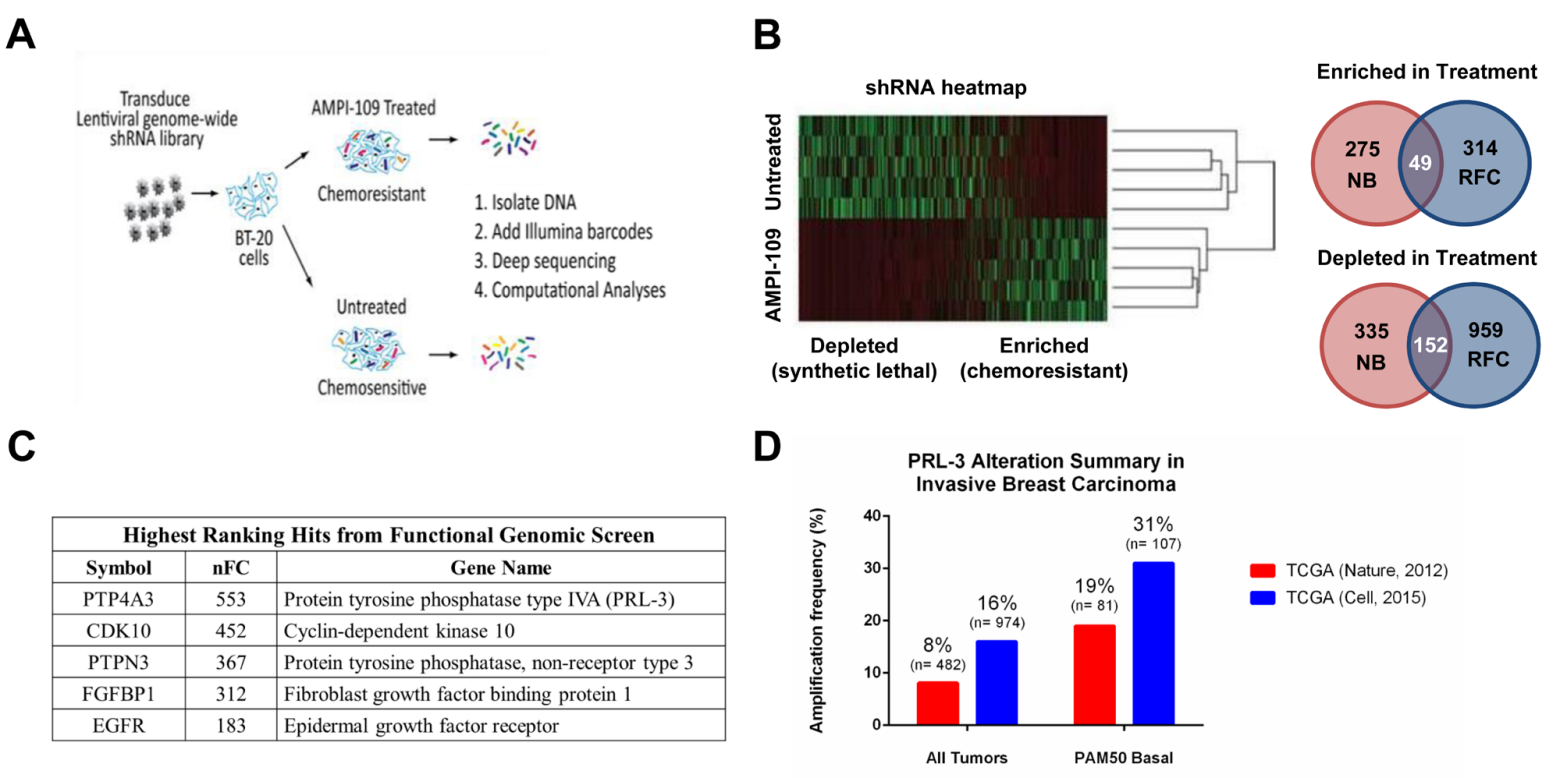
Functional genetic screen with AMPI-109 identifies PRL-3 amplification in invasive basal breast cancers **A.** Schematic representation of the experimental work flow of the functional genomic screen. BT-20 cells were transduced with a genome-wide shRNA library and treated with AMPI-109 (750 nM) or left untreated. After 5 passages the shRNA tag sequences were recovered and quantified by deep sequencing. Data were analyzed by complementary computational analyses generating lists of mediators of chemoresistance and chemosensitivity to AMPI-109. **B.** Heatmap of shRNA tag representation in AMPI-109-treated and untreated samples. Right panel: Two analysis strategies (NB, negative binomial and RFC, relative fold change) implicated 2,084 genes that modulate the effects of AMPI-109. 201 genes were identified by combined analysis strategies. **C.** Table highlighting highest ranking gene hits enriched by AMPI-109 treatment by normalized fold change (nFC). **D.** Bar graph depicting level of PRL-3 amplification in TCGA Nature, 2012 cohort (red) and TCGA Cell, 2015 dataset (blue) for patients with invasive breast cancer (all tumors) and those with invasive basal breast cancer as determined by the PAM50 signature (PAM50 Basal).

To identify non-VDR proteins involved in the cellular response to AMPI-109, and towards identifying signaling patterns unique to TNBC, we performed a genome-wide, functional genetic screen in the TNBC cell line BT-20 (Figure 4A). BT-20 cells were chosen for the screen because they were the most sensitive cell line in response to AMPI-109 (Table 1). Furthermore, we conducted the screen using a dose of AMPI-109 that was chosen to be significantly higher than the approximate IC_50_ dose in order to maximize the stringency of the screen and reduce the potential for false positives. We detected 13,303 unique shRNAs representing 8,628 genes after filtering reads with low representation (neither condition having at least 3 replicates with count values). The relative representation of the shRNAs were then compared, with the expectation that shRNAs under-represented after AMPI-109 treatment correspond to genes that confer chemosensitivity when inhibited (synthetic lethal), while shRNAs over-represented correspond to genes that confer chemoresistance. To identify “hits” from the screen, we applied two statistical analysis strategies to identify candidate genes for subsequent analysis and validation: 1) a negative binomial model with correction for multiple comparisons [19] and 2) a model requiring redundancy of shRNAs targeting a gene with a minimum 3-fold change in relative representation [20]. We surmised that genes common between the two analyses would be enriched for true positives and concentrated subsequent efforts on this overlapping list. Unsupervised clustering by Spearman's rank correlation with complete linkage demonstrated that replicates clustered based on treatment conditions (Figure 4B), indicating that enrichment or depletion of shRNAs was not stochastic, but due to AMPI-109 treatment. The analyses implicated 2,084 potential genes with 201 identified by both analysis methods (Figure 4B).

We then queried the overlapping gene set obtained from the shRNA screen against the Memorial Sloan-Kettering Cancer Center cBio Cancer Genomics Portal to determine the clinical significance of the genes identified. The highest ranking hit from our screen, *PTP4A3* (encoding PRL-3) (Figure 4C), was amplified or up regulated in approximately 8-16% of all invasive breast cancers between two TCGA datasets [8, 21] (Figure 4D). Amplification or up regulation of PRL-3 in invasive basal breast cancers, however, which includes TNBCs, ranged from 19-31% of cases based on the cohort examined (Figure 4D). These data suggested that PRL-3 levels may be higher in the breast cancer subtypes where AMPI-109 shows growth inhibitory activity. We therefore focused on the role of PRL-3 expression and activity on TNBC growth.

### PRL-3 knock down and expression of catalytically impaired PRL-3 inhibits TNBC cell growth and confers partial resistance to AMPI-109

PRL-3 is a dual-specificity protein tyrosine phosphatase that has been reported to be overexpressed in a number of cancer types including colorectal, gastric, ovarian, lung, liver and breast cancer [22-33]. Studies have reported roles for PRL-3 in modulating the cell cycle, promoting survival, and supporting tumor angiogenesis [34-40], but none to our knowledge have examined the phenotypic consequences of modulating PRL-3 expression or activity in TNBC cell lines.

**Figure 5:**
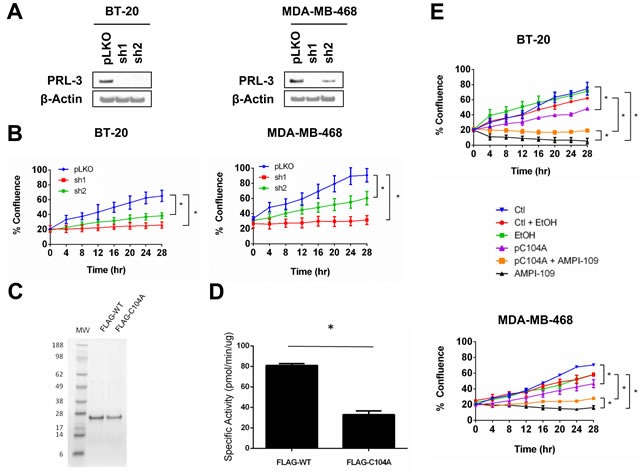
PRL-3 knock down and expression of catalytically impaired PRL-3 results in reduced growth of TNBC cells **A.** Immunoblot analysis of PRL-3 levels following transduction of BT-20 (left) and MDA-MB-468 (right) cells with lentiviral vectors expressing PRL-3 shRNAs (sh1 and sh2). pLKO is non-silencing control shRNA. **B.** Real time kinetic monitoring of cellular proliferation of BT-20 (left) and MDA-MB-468 (right) TNBC cells with non-silencing control shRNA (pLKO; blue) and PRL-3 shRNAs (sh1; red, sh2: green). **C.** SDS-PAGE analysis of FLAG-tagged recombinant wild type PRL-3 (FLAG-WT) and PRL-3 C104A (FLAG-C104A). Numbers indicate molecular weights (MW) in kilodaltons. **D.** The enzymatic activity of FLAG-tagged recombinant proteins was determined by *in vitro* phosphatase assay. **E.** BT-20 (top) and MDA-MB-468 (bottom) cells were transiently transfected with pC104A mammalian expression vector (pC104A; purple) or empty vector (Ctl; blue) 24 hrs prior to plating in IncuCyte. Cells were treated with ethanol vehicle control (EtOH; green) or 100 nM AMPI-109 (AMPI-109; black). Transfection control cells treated with ethanol vehicle (Ctl + EtOH; red) and pC104A cells treated with AMPI-109 (pC104A + AMPI-109; orange) are also depicted. Cellular proliferation measured by real time kinetic monitoring of plate confluence. * = *p*-value < 0.05 as determined by Student t test on last time-point.

We examined the role of PRL-3 in proliferation of TNBC cells by knocking down PRL-3. We used two shRNA sequences that were predicted by the Genetic Perturbation Platform of the Broad Institute to specifically target PRL-3 transcripts, but not the closely related family members PRL-1 and PRL-2, and observed significant knock down of PRL-3 protein in two TNBC cell lines (Figure 5A). Importantly, we verified knock down specificity for the PRL-3 shRNAs against both PRL-1 and PRL-2 by qRT-PCR. Both PRL-3 shRNAs (sh1 and sh2) exerted specific knock down action on PRL-3 and did not reduce RNA levels of either PRL-1 or PRL-2 (data not shown). In both lines, knock down of PRL-3 significantly impaired TNBC cellular proliferation (Figure 5B).

We also sought to determine the impact of impairing PRL-3 phosphatase activity to determine whether loss of activity could phenocopy the effect of knocking down PRL-3. It has been previously reported that mutation of the enzymatic nucleophile cysteine to serine or alanine abolishes phosphatase activity [41]. We therefore conducted site-directed mutagenesis and changed the coding sequence of the PRL-3 cDNA to result in a substitution of Cys^104^ to an alanine (C104A). To confirm that C104A results in a catalytically impaired phosphatase, we expressed and purified FLAG-tagged wild type (FLAG-WT) and C104A (FLAG-C104A) from E. coli (Figure 5C) and performed phosphatase assays using para-nitrophenyl phosphate (p-NPP) as substrate. It has been previously reported that a closely related PRL, PRL-1, is enzymatically active in hydrolyzing p-NPP to p-nitrophenol under reducing conditions [22]. Mutation of Cys^104^ to alanine significantly impaired, but did not completely abolish, the catalytic activity of PRL-3 (Figure 5D).

After confirming reduced activity of recombinant C104A phosphatase, we next examined the phenotypic consequences of expressing the catalytically impaired form of PRL-3 *in vivo*. We transiently transfected BT-20 and MDA-MB-468 cells with an expression vector containing the cDNA for C104A (pC104A). Interestingly, expression of C104A closely phenocopied PRL-3 knock down (Figure 5E), suggesting that forced expression of a catalytically impaired form of PRL-3 in TNBC cells may result in a dominant negative form of PRL-3.

In an effort to identify a preliminary relationship between PRL-3 and AMPI-109 mediated TNBC cell growth inhibition, we examined the consequences of treating pC104A transfected cell lines with AMPI-109, reasoning that C104A would either synergize with or confer resistance against AMPI-109 action. We observed partial resistance to AMPI-109 mediated inhibition of cell proliferation in cell lines transfected with pC104A (Figure 5E), suggesting PRL-3 may be involved in a signaling axis that is required for AMPI-109 action (BT-20 cells at 28 hours: Control = 92%, AMPI-109 = 68%; MDA-MB-468 cells at 28 hours: Control = 72%, AMPI-109 = 52%). This effect appears to be specific for PRL-3 because knock down of PRL-1 or PRL-2 did not confer any degree of resistance to AMPI-109. We are currently dissecting the mechanistic relationship between PRL-3 and AMPI-109 and how each of these affect TNBC growth potential.

### PRL-3 modulation and treatment with AMPI-109 inhibit TNBC cell migration and invasion

**Figure 6:**
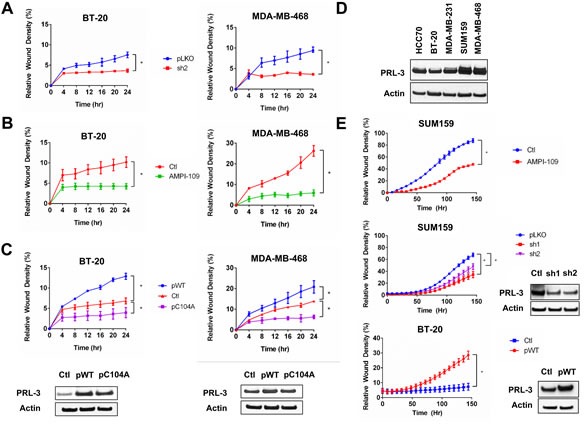
Knock down of PRL-3, AMPI-109 treatment and forced expression of catalytically impaired PRL-3 inhibit TNBC cell migration and invasion **A.** Quantification of wound closure in non-silencing control (pLKO; blue) and PRL-3 knock down (sh2; red) BT-20 and MDA-MB-468 cells as assessed by real time kinetic monitoring of relative wound density. **B.** BT-20 and MDA-MB-468 cells were treated with 100 nM AMPI-109 (AMPI-109; green) or ethanol vehicle control (Ctl; red) and cellular migration measured by scratch wound assay. **C.** BT-20 and MDA-MB-468 cells were transiently transfected overnight with mammalian expression vectors for wild type PRL-3 (pWT; blue), catalytically impaired PRL-3 (pC104A; purple) or empty vector (Ctl; red) and cellular migration measured by scratch wound assay. **D.** Immunoblot comparing PRL-3 protein levels across several TNBC cell lines. **E.** Real-time kinetic monitoring of cellular invasion through Matrigel Matrix in SUM159 and BT-20 cells treated with 100 nM AMPI-109 (top panel; red), stably expressing two different shRNA clones to knock down PRL-3 (middle panel; red and purple) or BT-20 cells transiently transfected to overexpress wild type PRL-3 (bottom panel; red). Invasive score was determined by the ability of cells to invade through a Matrigel wound (Relative Wound Density). Inset panels show PRL-3 levels by immunoblot analysis. * = *p*-value < 0.05 as determined by Student t test on last time-point.

Because TNBCs have high metastatic potential, we determined the effects of AMPI-109 treatment, PRL-3 expression and PRL-3 catalytic activity on cell migration and invasion. We first measured migration of BT-20 and MDA-MB-468 cells following PRL-3 knock down. After adjusting for differences in cell proliferation by nuclear count, knock down of PRL-3 almost entirely blocked the ability of these cells to migrate as early as 4 hours in both cell lines (Figure 6A). AMPI-109 also strongly inhibited the migration of BT-20 and MDA-MB-468 cells (Figure 6B). Additionally, forced expression of C104A in BT-20 and MDA-MB-468 cells decreased their migratory rates, providing further evidence for a dominant negative function of C104A in TNBC cells (Figure 6C). Conversely, we observed that overexpression of wild type PRL-3 significantly increased migration (Figure 6C).

Because MDA-MB-468 cells express high levels of PRL-3 but do not invade significantly in Matrigel *in vitro*, we chose to examine the consequences of treating with AMPI-109 and knocking down PRL-3 in SUM159 TNBC cells, which exhibit high levels of PRL-3 and are invasive in Matrigel *in vitro* (Figure 6D). Treatment with AMPI-109, or knock down of PRL-3 significantly impaired the ability of SUM159 TNBC cells to invade through Matrigel (Figure 6E). Conversely, BT-20 cells have lower levels of PRL-3 compared to MDA-MB-468 and SUM159 cells. We therefore examined the consequence of overexpressing wild-type PRL-3 in promoting the invasive potential of these cells. We observed that BT-20 cells exhibiting PRL-3 overexpression could invade through Matrigel more rapidly and to a higher degree relative to control cells (Figure 6E). Taken together, our data support evidence in colorectal cancer, where high expression of PRL-3 is implicated in driving cell motility, invasion and metastasis [27, 28, 30]. These data strongly demonstrate that PRL-3 also plays a pivotal role in promoting TNBC cell migration and invasion, critical steps for cancer cell metastasis.

### PRL-3 expression positively associates with the TNBC subtype, regional and distant metastases

**Figure 7:**
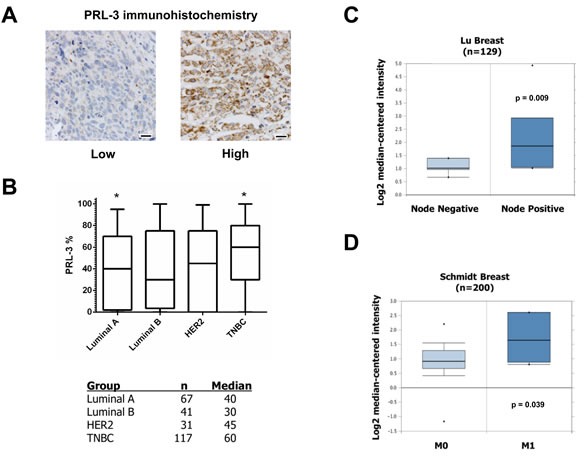
PRL-3 expression is increased in TNBC **A.** Examples of PRL-3 staining scored as low or high, 20 μm scale bars. **B.** Whisker plot and table of PRL-3 expression across different breast cancer subtypes. * = *p*-value (< 0.008) based on post hoc Dunn's test from Kruskal-Wallis non-parametric ANOVA. (C and D) Differential PRL-3 mRNA expression in two human breast cancer datasets examining correlation to regional disease and visceral metastases.

Our data implicates an oncogenic role for PRL-3 in TNBC by 1) providing a growth advantage to cells expressing PRL-3 and 2) enhancing TNBC cell migratory and invasion ability. To examine the *in vivo* relevance of PRL-3 expression, we evaluated PRL-3 protein expression in human breast cancers and its relationship to several clinicopathologic variables. Immunohistochemical analysis of archival human breast tissue was carried out for PRL-3 (Figure 7A). Using the percentage of tumor cells positive for PRL-3 among the four subtypes, we determined that PRL-3 expression was significantly higher in TNBC *versus* luminal A breast cancer (*p* = 0.008, Figure 7B). We also observed a significantly higher percentage of PRL-3 positive tumor cells in patients who had lymph node metastases (regional disease) at the time of diagnosis (*n* = 48, 66%) as compared with patients whose nodes were tumor free (*n* = 69, 45%; *p* = 0.024).

We augmented our immunohistochemical analyses through mining of publicly available microarray tumor data using Oncomine. We corroborated higher PRL-3 mRNA expression in TNBC *versus* other breast cancer subtypes, including non-tumorigenic tissue, and PRL-3 association with regional disease in the Lu Breast cohort (Figure 7C) [42]. Multiple other cohorts also showed an association between PRL-3 and regional disease (Table 2). We also determined a positive correlation with metastatic (M1) *versus* visceral metastases-free disease (M0) in the Schmidt Breast cohort (Figure 7D) [43]. Finally, multiple datasets revealed significant associations between increased PRL-3 expression in cancerous *versus* normal tissue, invasive ductal breast carcinoma compared to non-invasive disease, increased tumor grade, stage and development of metastases at 1 and 3 year intervals following diagnosis (Table 2). Collectively, these data support our *in vitro* findings that PRL-3 acts as an oncogenic mediator to drive aggressive phenotypes in breast cancer.

**Table 2:** PRL-3 mRNA expression correlates with multiple clinicopathologic variables of aggressive breast cancer

	N	Cancer vs. Normal FC	P-value
Richardson Breast 2	47	2.288	2.81e^−6^
	N	IBC vs. normal FC	P-value
Curtis Breast	2,136	1.369	1.13e^−4^
TCGA Breast	593	1.949	6.87e^−15^
Gluck Breast	158	1.491	4.00e^−3^
	N	IDC vs. normal FC	P-value
Curtis Breast	2,136	1.391	1.54e^−37^
TCGA Breast 2	1,602	1.27	3.60e^−72^
Turashvili Breast	30	2.441	2.00e^−03^
	N	TNBC vs. other subtypes FC	P-value
Richardson Breast 2	47	2.043	1.50e^−2^
Minn Breast 2	121	1.274	1.03e^−04^
Kao Breast	327	1.427	5.00e^−3^
Chin Breast	118	1.485	5.00e^−3^
KordeBreast	61	1.298	3.00e^−2^
Bonnefoi Breast	160	1.186	1.70e^−2^
	N	Grade 3 vs. 2 FC	P-value
Desmedt Breast	198	1.931	1.00e^−2^
Nik-Zainal Breast	21	1.378	4.00e^−3^
	N	Lymph node positive vs. negative FC	P-value
Stickeler Breast	57	1.901	3.10e^−2^
Lu Breast	129	2.028	9.00e^−3^
	N	5 year recurrence FC	P-value
Finak Breast	59	1.24	2.30e^−2^
Loi Breast 3	77	1.343	3.20e^−2^
Ma Breast 3	60	1.325	2.30e^−2^
	N	Metastasis at 1 year FC	P-value
Schmidt Breast	200	1.509	3.90e^−2^
	N	Metastasis at 3 years FC	P-value
Symmans Breast 2	103	1.393	5.00e^−2^
Bos Breast	204	1.264	9.00e^−3^
Kao Breast	327	1.274	8.00e^−3^

Left column, Oncomine dataset examined. N = Sample size in analysis. IBC = Invasive Breast Cancer. IDC = Invasive Ductal Carcinoma. TNBC = Triple-Negative Breast Cancer. FC = Fold Change in PRL-3 expression. *P* values computed by Pearson *r* test.

## DISCUSSION

TNBCs are among the most aggressive breast cancer subtypes and are associated with a higher risk of metastasis and death as compared to other breast cancer subtypes [3, 44, 45]. While a molecular explanation for this higher risk associated with poor outcome remains unknown, it is likely due, in part, to signaling pathways governing TNBC cell migration and invasion that enhance TNBC metastatic potential.

Here, we investigated the pre-clinical activity of AMPI-109 in TNBC, and used AMPI-109 as a tool in a proof-of-principle genome-wide functional genetic screen aimed at uncovering molecular aberrations peculiar to TNBC. Among non-tumorigenic and tumorigenic mammary cell lines tested, we observed almost complete specificity of AMPI-109 for inhibition of growth, migration, invasion and promotion of apoptosis in TNBC cell lines. These results are of significance because they suggest AMPI-109 may have therapeutic potential in TNBC. Ongoing pre-clinical *in vivo* studies will evaluate this hypothesis. To understand whether the specificity of AMPI-109 against TNBC can reveal unique alterations in TNBC, we performed a genome-wide shRNA screen in BT-20 TNBC cells treated with AMPI-109. Through the analysis of shRNAs enriched in the AMPI-109 treated arm, we identified the dual-specificity phosphatase, PRL-3 as a potential mediator of AMPI-109 action in TNBC cells. Significantly, other top ranking hits from our screen, such as CDK10, FGFBP1 and EGFR, have also been implicated as being overexpressed or pro-tumorigenic in the context of breast cancer, lending robust utility for our genome-wide screen with AMPI-109 in identifying aggressive, cancer-related markers.

Our data demonstrate that PRL-3 knock down or impairment of PRL-3 activity phenocopies but does not synergize with AMPI-109 in impairing TNBC cell growth, migration and invasion. The exact mechanistic relationship between AMPI-109 and PRL-3, including the possibilities that AMPI-109 directly targets PRL-3, alters its transcriptional or translational levels or affects PRL-3 protein stability, remains an active area of investigation for our laboratory. We propose it unlikely that PRL-3 is the sole “modifier” of AMPI-109 activity, as PRL-3 appears to be expressed in non-TNBC subtypes as evidenced by immunoblot in non-TNBC cell lines and our retrospective IHC analyses. Therefore, additional modifiers for AMPI-109 or other PRL-3 pathway associated effectors likely exist and the identification of these molecules is also actively ongoing in our laboratory.

The identification of a phosphatase as an oncogenic driver was initially somewhat surprising. Phosphatases are typically regarded to be the “off” switches of molecular signaling pathways that drive cell growth and proliferation. As a result, much work in translational oncology has focused on understanding and targeting kinases, the “on” switches, with less attention being paid to phosphatases. Recently, however, there have been extensive efforts to elucidate the roles of phosphatases in the development and progression of various cancers, including breast cancer [22-25].

Perhaps the most characterized role of PRL-3, extensively studied in colorectal cancer, is the promotion of cell migration, invasion and metastasis. One proposed model by which this is thought to occur is PRL-3 mediated downregulation of PTEN, leading to activation of PI3K/Akt signaling and induction of epithelial to mesenchymal transition [46]. Further studies propose direct interaction between PRL-3 and ezrin and/or integrin α1 and β1 to alter focal adhesion signaling pathways responsible for promoting cell restructuring and migration [47-49]. However, we did not observe changes in ezrin phosphorylation after PRL-3 modulation in BT-20 and MDA-MB-468 TNBC cells (data not shown). One possible explanation for this finding may be that ezrin is not a PRL-3 substrate in TNBC cells. We did, however, observe lower levels of Akt phosphorylation after PRL-3 knock down in MDA-MB-231 TNBC cells (data not shown). To understand the molecular mechanism behind the observed oncogenic functions of PRL-3 in TNBC, we are currently examining the cellular signaling pathways modulated by PRL-3 that promote growth, migration and invasion of TNBC cells. Understanding these pathways will be critical to expanding our knowledge of how PRL-3 promotes aggressive TNBC phenotypes. For example, MDA-MB-468 TNBC cells which express high levels of PRL-3, but do not invade through Matrigel *in vitro*, may possess additional biomarkers, such as matrix metalloproteinases or focal adhesion components, which could be used to further stratify the invasive potential of TNBC cells beyond PRL-3.

In addition, previous studies have associated high PRL-3 expression with poor prognoses in node-positive TNBC and other subtypes including c-Erb-B overexpressing breast tumors [33, 50], but very little mechanistic evidence implicates a role for PRL-3 in driving breast cancer progression. Our finding that PRL-3 is overexpressed in invasive human basal tumors, which include TNBCs, in parallel with our evidence demonstrating knock down or catalytic impairment of PRL-3 mimic the effect of AMPI-109 on inducing growth, migratory and invasion blockade, imply that PRL-3 may be a critical component behind the aggressive nature of TNBCs. In concordance with the studies presented herein, another study has preliminarily reported that PRL-3 is critical for cell growth of TNBC [51]. Interestingly, overexpression of PRL-3 in BT-20 and MDA-MB-468 TNBC cells does not lead to additional growth advantages. These data suggest that PRL-3 expression levels in these cells already confers the maximal growth promoting capabilities of PRL-3. However, overexpression of PRL-3 does partially abrogate the antiproliferative properties of AMPI-109 (data not shown).

In summary, we have identified AMPI-109 as a pre-clinical agent active against TNBC cell lines and have identified the oncogenic phosphatase PRL-3, as a modulator of TNBC growth, migration and invasion. Our data warrant further investigations into the mechanistic relationship between AMPI-109 and PRL-3 and merit additional studies exploring the oncogenic PRL-3 mechanisms of action in TNBC in an effort to better understand the aggressive biological nature of this disease.

## MATERIALS AND METHODS

### Materials

AMPI-109 was synthesized as previously described [15]. PRL-3 cDNA expression vector was purchased from Origene (Cat. # SC308739).

### Cell culture, immunoblot analysis and transfection

Cell lines were obtained from the University of Colorado Cancer Center Tissue Culture Shared Resource. All cell lines were authenticated by short tandem repeat DNA profiling performed by the University of Colorado Cancer Center DNA Sequencing and Analysis Core. Western blot analysis was conducted as previously described [16]. c-PARP (Cat. # 5625, Cell Signaling), VDR (Cat. # 12550, Cell Signaling), PRL-3 (Cat. # ab82568, Abcam), β-actin (Cat. # A5441, Sigma Aldrich). Transfections were carried out using Mirus TransIT LT1 transfection reagent according to manufacturer's instructions (Mirus Bio).

### Production of wild type and C104A FLAG-tagged recombinant PRL-3

Wild type and C104A cDNAs were amplified from the respective mammalian expression vectors by PCR using the oligonucleotides: 5′-T*AAGCTTA*TGGCTCGGATGAACCGCCCGGCCCG-3′ and5′-GAT*CTCGAG*CTACATAACGCAGCACCGGGTCTTG-3′. Products were ligated into pCR2.1-TOPO (Invitrogen) for DNA sequencing. Following confirmation of sequences, wild type and C104A cDNAs were excised with HindIII and XhoI and ligated into bacterial expression vector pFLAG-MAC (Sigma) that had been digested with HindIII and XhoI. Expression vectors were transformed into the E. coli strain BL21. Production and purification of FLAG-tagged proteins was carried out according to the manufacturer's instructions (Sigma).

### Site-directed mutagenesis

Cys^104^ to alanine substitution in the PRL-3 cDNA was created using the Quickchange Site-Directed mutagenesis kit (Agilent) and confirmed by DNA sequencing. Oligonucleotides used were: 5′-GCGTGGCTGTGCACGCCGTGGCGGGCCTGGG-3′ and 5′-CCCAGGCCCGCCACGGCGTGCACAGCCACGC-3′.

### Lentivirus

Individual pLKO.1 lentiviral shRNA clones and the MISSION™ human genome-wide shRNA library were purchased from the University of Colorado Cancer Center Functional Genomics Shared Resource. The RNAi Consortium identifiers used in this study were TRCN0000010661 (PRL-3 sh1), TRCN0000355597 (PRL-3 sh2), TRCN0000019506 (VDR3) and TRCN0000276542 (VDR8). Transduced cells were selected in medium containing 2.5 μg/mL puromycin.

### Genome-wide functional genetic screening

BT-20 cells were transduced with the MISSION™ human genome-wide shRNA library (Sigma), and shRNA expressing cells selected in puromycin (2.5 μg/mL). Cells were then split into 10 replicates: 5 were left untreated and 5 were treated with AMPI-109 (750 nM) for 5 passages. The shRNA tag sequences were isolated from the DNA using vector specific primers and quantified on an Illumina Genome Analyzer_IIx_ as previously described [19].

### Apoptosis assays

CellPlayer 96-well Kinetic Caspase-3/7 reagent was used according to manufacturer's instructions at 5 μM (Essen Bioscience). Cells were plated overnight and the following day treated with AMPI-109 (100 nM) or vehicle control. Cell Death Detection ELISA kit was used according to manufacturer's instruction as a complementary method for apoptosis detection (Cat. # 11 544 675 001, Roche).

### Cellular proliferation, migration and invasion assays

Cell proliferation was assessed by CellTiter 96 MTS Proliferation assay (Cat. # G109A, Promega) and IncuCyte Zoom Kinetic Live Cell Imaging (Essen BioScience). For MTS, samples were read at 490 nm in a Synergy 2 microplate reader (BioTek). Proliferation rates were determined using IncuCyte based on percentage of confluence over time. For migration, cells were plated in 96 well ImageLock Plates (Essen Bioscience). A scratch was made using a 96-pin WoundMaker (Essen Bioscience). For invasion, 96 well ImageLock Plates were coated overnight with 100 μg/mL Matrigel Basement Membrane Matrix (Cat. # 356231, Corning). The following day, Matrigel was aspirated and 25,000 cells were plated per well and allowed to adhere prior to making a scratch with the 96-pin WoundMaker. 50μL of Matrigel Matrix (8 mg/mL) was then added to the wells, and covered in 100 μL media containing the appropriate treatments (refer to figure legend for details). Migration and invasion were quantified using the “Relative Wound Density” metric generated by IncuCyte software.

### *In vitro* enzymatic assay

Purified, recombinant PRL-3 protein (500 μg) was added to phosphatase assay buffer (12.5 mM p-NPP, 50 mM Tris 7.4, 150 mM NaCl, and 5 mM DTT), in a final volume of 50 μL and incubated for 30 minutes at 30° C in 96 well plates. After 30 minutes, p-nitrophenol was determined at 405 nm using a Synergy 2 microplate reader (BioTek). Specific activity was calculated using an extinction coefficient of 1.78 × 10^4^ for p-NPP.

### Immunohistochemistry

Archival formalin fixed paraffin embedded (FFPE) tumors from 117 TNBCs, 67 Luminal A, 43 Luminal B and 29 HER2 patients diagnosed at the Massachusetts General Hospital between 1976 and 1993 were used. All specimens were covered by an IRB approved protocol. Tumor size, patient age at diagnosis and nodal status was determined by review of pathology reports. IHC methods for ER, PR and HER2 are previously reported [52]. FFPE sections were utilized for IHC using standard methods. PRL-3 antibody previously validated for IHC (1:4000; Cat. # ab82568 (PTP4A3), Abcam) was used as a primary reagent at 4° C. Two pathologists (A.D.T. and S.M.E.) reviewed the entire slide and determined the percentage and intensity of tumor cells that were positive for cytoplasmic PRL-3 staining.

### Microarray and statistical analysis

Microarray datasets from breast tumors and respective statistics were obtained from Oncomine (www.oncomine.com, Oct 2015, Thermo Fisher Scientific, Ann Arbor, MI). Significance was calculated using the Student t test in GraphPad Prism version 6.04 software. Bioinformatic statistical analysis of the functional genomic screen was carried out as previously described [19]. To determine differences in PRL-3 expression in breast cancer subtypes, non-parametric Kruskal-Wallis ANOVA and post hoc Dunn's test was conducted to determine which tumor types were significantly different from each other. Analyses were conducted using SAS (ver 9.4, SAS Institute) or GraphPad Prism. Tests were two-sided with significance set at *p* < 0.05.
